# Optimal strategies for treatment discontinuation in MOG antibody-associated disease

**DOI:** 10.1093/brain/awag006

**Published:** 2026-02-04

**Authors:** Wei Z Yeh, Anna Francis, Sarah Cooper, Waqar Rashid, Roswell Martin, Jeremy Hobart, Christopher Halfpenny, Victoria Williams, Eoin O’Sullivan, Cheryl Hemingway, Yael Hacohen, Ruth Dobson, Patrick Waters, Srilakshmi M Sharma, Helmut Butzkueven, Ruth Geraldes, Sithara Ramdas, Maria Isabel Leite, Jacqueline Palace

**Affiliations:** Nuffield Department of Clinical Neurosciences, University of Oxford, Oxford OX3 9DU, UK; Department of Clinical Neurology, John Radcliffe Hospital, Oxford University Hospitals NHS Foundation Trust, Oxford OX3 9DU, UK; Department of Neuroscience, School of Translational Medicine, Monash University, Melbourne, Victoria 3004, Australia; Department of Neurology, Alfred Health, Melbourne, Victoria 3004, Australia; Nuffield Department of Clinical Neurosciences, University of Oxford, Oxford OX3 9DU, UK; Department of Clinical Neurology, John Radcliffe Hospital, Oxford University Hospitals NHS Foundation Trust, Oxford OX3 9DU, UK; Department of Neurology, University Hospitals Sussex National Health Service Foundation Trust, Brighton BN2 5BE, UK; Department of Neurology, St George’s University Hospitals National Health Service Foundation Trust, London SW17 0QT, UK; Department of Neurology, Gloucestershire Hospitals National Health Service Foundation Trust, Gloucestershire GL1 3NN, UK; Department of Neurology, University Hospitals Plymouth National Health Service Foundation Trust, Plymouth PL6 5FP, UK; Department of Neurology, University Hospitals Southampton National Health Service Foundation Trust, Southampton SO16 6YD, UK; Department of Neurology, Guy’s and St Thomas’ National Health Service Foundation Trust, London SE1 7EH, UK; Eye Department, King’s College Hospital, London SE5 9RS, UK; Department of Paediatric Neurology, Great Ormond Street Hospital for Children, London WC1N 3BH, UK; Department of Paediatric Neurology, Great Ormond Street Hospital for Children, London WC1N 3BH, UK; Preventive Neurology Unit, Wolfson Institute of Preventive Medicine, Queen Mary University London, London EC1M 6BQ, UK; Royal London Hospital, Barts Health National Health Service Foundation Trust, London E1 1BB, UK; Nuffield Department of Clinical Neurosciences, Oxford Autoimmune Neurology Diagnostic Laboratory, University of Oxford, Oxford OX3 9DU, UK; Oxford Eye Hospital, John Radcliffe Hospital, Oxford University Hospitals NHS Foundation Trust, Oxford OX3 9DU, UK; Kennedy Institute for Rheumatology, University of Oxford, Oxford OX3 7FY, UK; Department of Neuroscience, School of Translational Medicine, Monash University, Melbourne, Victoria 3004, Australia; Department of Neurology, Alfred Health, Melbourne, Victoria 3004, Australia; Nuffield Department of Clinical Neurosciences, University of Oxford, Oxford OX3 9DU, UK; Department of Clinical Neurology, John Radcliffe Hospital, Oxford University Hospitals NHS Foundation Trust, Oxford OX3 9DU, UK; Nuffield Department of Clinical Neurosciences, University of Oxford, Oxford OX3 9DU, UK; Department of Clinical Neurology, John Radcliffe Hospital, Oxford University Hospitals NHS Foundation Trust, Oxford OX3 9DU, UK; Nuffield Department of Clinical Neurosciences, University of Oxford, Oxford OX3 9DU, UK; Department of Clinical Neurology, John Radcliffe Hospital, Oxford University Hospitals NHS Foundation Trust, Oxford OX3 9DU, UK; Nuffield Department of Clinical Neurosciences, University of Oxford, Oxford OX3 9DU, UK; Department of Clinical Neurology, John Radcliffe Hospital, Oxford University Hospitals NHS Foundation Trust, Oxford OX3 9DU, UK

**Keywords:** myelin oligodendrocyte glycoprotein antibody-associated disease, treatment, discontinuation, clinical outcome

## Abstract

The relapse risk following discontinuation of immunomodulatory treatment in myelin oligodendrocyte glycoprotein antibody-associated disease (MOGAD) is unknown. Evidence suggests that ‘at least’ 3 months of oral corticosteroids reduces relapse risk after a single attack, and that it may be possible to stop maintenance treatment in relapsing stable disease, but the optimal duration of treatment has yet to be defined. We therefore investigated relapse outcomes following discontinuation of maintenance treatment.

We conducted a cohort study of MOGAD patients seen in the Oxford Neuromyelitis Optica Highly Specialised Service between January 2010 and May 2025. Patients with MOGAD who had at least 12 months of follow-up and who commenced and then discontinued maintenance treatment were included. Associations of factors including treatment duration before discontinuation, disease course at discontinuation (after a single attack/monophasic or relapsing course) and MOG IgG1 status on live cell-based assay were investigated. The primary outcome was time-to-relapse following treatment discontinuation. Cox regression was used.

We included 190 MOGAD patients with 236 discontinued treatment intervals. There were 150 (63.6%) discontinuations after a single attack and before a first relapse in patients with monophasic disease, and 86 (36.4%) discontinuations in patients with relapsing disease. Most patients used corticosteroids alone [84.7% of immunomodulatory treatment (IT) intervals], and non-steroid ITs were used in 15.2% of IT intervals, either alone or in combination with steroids. Post-discontinuation relapse occurred after 92 (39.0%) discontinuations, at a median time of 5.4 (interquartile range 1.4–20.1) months after treatment cessation. Those who relapsed were more likely to have a relapsing course at the time of discontinuation (50% versus 27.8%, *P* = 0.001) and a positive/low-positive pre-discontinuation MOG IgG1 result (89.8% versus 71.5%, *P* = 0.005). In a multivariable analysis, a relapsing course at the time of discontinuation was associated with an elevated relapse risk (hazard ratio 1.95, 95% confidence interval 1.25–3.06, *P* = 0.003). Overall, prolonged treatment beyond 3 months before discontinuation significantly reduced relapse risk. Optimal treatment durations were estimated to be 10–18 months for patients treated after their first attack and 20–30 months for relapsing patients, after which treatment discontinuation could be considered in patients who were relapse-free on treatment.

Identifying the relapse risk when discontinuing maintenance immunomodulatory treatment in MOGAD should aid management decisions in patients presenting with their first attack and also in those on longer-term treatment for relapsing disease. Our findings, from a cohort predominantly treated with steroids, provide evidence to inform joint decision-making for stable patients who are considering treatment cessation.

## Teaser

Yeh *et al.* examine the risk of relapse following discontinuation of maintenance immunotherapy in a large cohort of patients with MOGAD. They estimate the optimal duration of treatment before discontinuation can be considered, comparing patients treated after a first attack with those who have relapsing disease.

**See Sechi and Cortese. (https://doi.org/10.1093/brain/awag278) for a scientific commentary on this article.**

## Introduction

Myelin oligodendrocyte glycoprotein antibody-associated disease (MOGAD) is a recently defined inflammatory disease of the CNS.^1^ Treatment is empirical, using immunomodulatory treatments (ITs) that include corticosteroids and steroid-sparing agents such as azathioprine, mycophenolate and intravenous immunoglobulin (IVIG). MOGAD appears highly corticosteroid-responsive, with observational data supporting an oral prednisolone course of at least 3 months to delay time-to-relapse from onset.^2,3^

The disease course of MOGAD may be monophasic or relapsing. A relapsing course is reported in 30%–50% of patients within the first 4 years from onset.^4,5^ This is in contrast with aquaporin-4 antibody-positive neuromyelitis optica spectrum disorder (NMOSD), in which a relapsing course is seen in approximately 90% of cases.^6-8^ In a cohort of untreated MOGAD patients, relapse rates appeared highest soon after disease onset and reduced over time.^2,4,9^

Many expert centres currently use different treatment paradigms for managing patients who have had a single attack and those who have relapsed. After a single attack, patients are often treated with a short course of oral corticosteroids for 3–6 months following acute attack management, although this can vary. Conversely, in relapsing patients, longer-term treatment with either corticosteroids or steroid-sparing agents is used. Given data showing a reduction in relapse rates over time, many MOGAD experts now consider withdrawal of immunosuppression after several years of disease stability.^4,9^

There is currently limited evidence to guide the optimal length of time on treatment before discontinuation in those presenting with their first attack and in those who have relapsed. Studies that included on-treatment time and used minimum treatment period(s) suggested that at least 3 months of treatment can delay time-to-relapse, but how long beyond 3 months is not known.^2,3,10^ Only one study has previously investigated time-to-relapse after IT discontinuation, and it included treatment-length categories of up to 6 months.^2^ Data are required to inform decisions around potential treatment cessation, and to help balance risks and benefits of immunosuppression. Therefore, we conducted a large single-service cohort study to investigate relapse outcomes after treatment discontinuation and identify predictors for post-discontinuation relapse.

## Materials and methods

### Study design and population

Patients fulfilling 2023 MOGAD criteria^1^ seen in the Oxford Neuromyelitis Optica Highly Specialised Service between January 2010 and May 2025 and who consented to participate in the Demyelinating Research Tissue Bank (approved by Oxford C Research Ethics Committee, reference 21/SC/0353) were assessed for inclusion. Data were extracted on 2 May 2025.

We conducted a retrospective cohort study using data that were prospectively collected upon service entry and actively databased at initial and subsequent reviews. Data included demographics, attack characteristics, treatment and serum MOG IgG1 results from live-cell-based assays.^11^ Patients were included if they had ≥12 months of follow-up and had received eligible maintenance IT (oral corticosteroids, azathioprine, mycophenolate, methotrexate, IVIG or monoclonal antibody therapies) that were discontinued.

The primary outcome was time-to-relapse after IT discontinuation. Relapse was defined as a new clinical attack >30 days after the prior attack. All relapses were adjudicated by one of two expert NMOSD consultants (J.P. or M.I.L.).

We categorized discontinuations by the patient’s disease course at the time of IT discontinuation. Disease course at time of IT discontinuation was referred to as ‘monophasic’ if discontinuation occurred after a single attack and before a first relapse, and as ‘relapsing’ if discontinuation occurred after any relapse. Acute treatment for an onset attack comprised high-dose methylprednisolone, plasma exchange and/or IVIG. The timing of acute treatment was classified as early (<5 days from attack), intermediate (5–14 days from attack) or late (>14 days from attack), based on categories used in a recent study that found an association between the timing of acute treatment for an onset attack and relapse risk.^12^

### Statistical analyses

Mann–Whitney U-test and Fisher’s exact test were used to compare baseline characteristics.

Univariable and multivariable Cox regression were used to investigate predictors of time-to-relapse following discontinuation. Robust standard errors were calculated with clustering by patient ID to account for multiple discontinuations contributed by some patients. Included covariates were determined *a priori* based on clinical relevance. These were: age at onset, sex, onset attack phenotype, timing of acute treatment at onset, IT types used, IT duration before discontinuation, disease course at discontinuation, number of relapses before discontinuation (in analysis of relapsing patients), and most recent MOG IgG1 status before discontinuation. Pre-discontinuation IT duration was modelled using restricted cubic splines with three knots to allow for non-linear associations with the outcome, and its effect on the outcome was compared with a reference of 3 months, based on cut-off points from prior studies.^2,3,10^ Baseline was the time of IT discontinuation. The Schoenfeld's global test was used to assess the proportional hazard assumption. Multiple imputation was used to account for missing data (the timing of acute treatment after the onset attack was missing in 1.1% of participants; pre-discontinuation MOG IgG1 status was missing for 16.9% of IT intervals) with 100 imputed datasets.

Analyses were performed for all patients with discontinuations, for discontinuations that occurred when the disease course was monophasic or relapsing at the time of discontinuation, respectively, and for an incident cohort defined as patients whose first MOG IgG-positive test was performed after the onset attack and before a first relapse. We estimated optimal treatment durations at which the relapse hazard first approached a minimum by visualizing the adjusted hazard ratio (HR) as a function of treatment duration. This was done separately for patients who discontinued treatment when the disease course was monophasic or relapsing, given differing relapse risks and treatment strategies. Based on prior studies showing the potential prognostic value of MOG IgG status,^13-15^ we conducted a sensitivity analysis that included the IT intervals for which MOG IgG1 status was available within 12 months of the IT discontinuation date.

A two-tailed *P* < 0.05 was considered significant. Analyses were performed in the R Statistical Environment (v4.4.1) with tidyverse, survival, mice and rms packages.^16-20^

## Results

### Cohort characteristics

We included 190 participants with MOGAD, contributing 236 discontinued IT intervals (Supplementary Fig. 1). Most participants contributed one discontinued IT interval (153/190, 80.5%), while 37/190 (19.5%) participants contributed up to three intervals (Table 1). The median age at disease onset was 31.5 years [interquartile range (IQR) 16.2–41.9], and 115 (60.5%) participants were female. At last follow-up, median disease duration was 4.9 years (IQR 2.5–8.2) and 86 (45.3%) had a relapsing course.

**Table 1:** Clinical characteristics of participants

Characteristics	Included participants (*n* = 190)
Age at onset, years, median (IQR; range)	31.5 (16.2–41.9; 1.4–77.2)
Sex, *n* (%)	
Female	115 (60.5)
Male	75 (39.5)
Race, *n* (%)	
White	150 (78.9)
Asian	17 (8.9%)
Black/African	4 (2.1)
Middle Eastern	3 (1.6)
American Indian and Alaskan Native	1 (0.5)
Hispanic	1 (0.5)
Mixed	6 (3.2)
Not known	8 (4.2)
Disease duration at last follow-up, years, median (IQR; range)	4.9 (2.5–8.2; 1.0–50.9)
Onset attack phenotype, *n* (%)	
Optic neuritis	108 (56.8)
Myelitis	31 (16.3)
ADEM or brain	18 (9.5)
Mixed	33 (17.4)
Acute management for onset attack, *n* (%)^a,b^	
Early (<5 days)	21 (11.1)
Intermediate (5–14 days)	68 (35.8)
Late (>14 days) or no treatment	99 (52.1)
Acute management for onset attack with methylprednisolone, *n* (%)	151 (79.5)
Acute management for onset attack with plasma exchange, *n* (%)	21 (11.1)
Acute management for onset attack with intravenous immunoglobulin, *n* (%)	9 (4.7)
CSF-restricted oligoclonal bands present, *n* (%)^c^	3 (3.0)
Incident cohort, *n* (%)	148 (77.9)
Disease course at last follow-up, *n* (%)	
Monophasic	104 (54.7)
Relapsing	86 (45.3)
Discontinued IT intervals included, *n* (%)	
1	153 (80.5)
2	27 (14.2)
3	10 (5.3)

ADEM = acute disseminated encephalomyelitis; IT = immunomodulatory treatment.

^a^Acute management includes high-dose methylprednisolone, plasma exchange and/or intravenous immunoglobulin.

^b^Acute management timing available for 188/190 (98.9%) participants.

^c^CSF oligoclonal band result available for 101/190 (53.2%) participants.

Of 236 discontinued intervals, 150 (63.6%) occurred during monophasic disease and 86 (36.4%) during relapsing disease (Table 2). Of 86 intervals discontinued during relapsing disease, the median number of relapses before discontinuation was 1 (IQR 1–2). The majority were relapse-free on treatment [229/236 (97%) IT intervals]. Most participants used only corticosteroids [200/236 (84.7%) IT intervals; 139/150 (92.7%) intervals were discontinued when the disease course was monophasic and 61/86 (70.9%) when the course was relapsing], whereas non-steroid ITs were used either alone or in combination with steroids in 36/236 (15.2%) IT intervals. Azathioprine and mycophenolate were the most commonly used non-steroidal ITs. Most discontinuations were planned (210/236, 89%). There were seven (3.0%) discontinuations due to adverse events.

**Table 2:** Characteristics of discontinued immunomodulatory treatment intervals

Characteristics	All discontinued IT intervals (*n* = 236)	No relapse post-discontinuation (*n* = 144)	Relapse post-discontinuation (*n* = 92)	*P-*value
Age at discontinuation, years, median (IQR; range)	35.4 (17.3–45.0; 1.5–79.0)	35.8 (20.8–45.6; 1.5–79.0)	35.0 (14.7–43.7; 2.9–72.9)	0.37
Disease duration at discontinuation, years, median (IQR; range)	1.3 (0.6–2.6; 0.03–44.7)	1.3 (0.8–2.3; 0.07–44.7)	1.1 (0.4–3.0; 0.03–30.2)	0.26
Disease course at discontinuation, *n* (%)				
Monophasic	150 (63.6)	104 (72.2)	46 (50)	0.001
Relapsing	86 (36.4)	40 (27.8)	46 (50)	
No. of relapses prior to discontinuation, median (IQR; range)	0 (0–1; 0–5)	0 (0–1; 0–5)	0.5 (0–2; 0–5)	<0.001
Relapse during IT interval, *n* (%)	7 (3.0)	2 (1.4)	5 (5.4)	0.16
Duration between last attack and discontinuation, median months, (IQR; range)	10.7 (3.8–17.0; 0.1–122.0)	13.4 (7.1–18.5; 0.9–91.8)	6.5 (1.7–14.6; 0.1–122)	<0.001
Duration of IT interval, months, median (IQR; range)	9.6 (2.9–15.6; 0.07–122.0)	11.8 (5.0–16.9; 0.3–85.7)	4.7 (1.0–13.0; 0.1–122)	<0.001
IT types used during IT interval, *n* (%)				
Corticosteroids only	200 (84.7)	125 (86.8)	75 (81.5)	0.54
Non-steroid IT only	13 (5.5)	7 (4.9)	6 (6.5)	
Both corticosteroids and non-steroid IT	23 (9.7)	12 (8.3)	11 (12)	
Non-steroid IT used, *n* (%)^a^				
Azathioprine	21 (58)	11 (57.9)	10 (58.8)	N/A
Mycophenolate	13 (36)	8 (42.1)	5 (29.4)	
IVIG	3 (8)	2 (10.5)	1 (5.9)	
Methotrexate	2 (6)	0 (0)	2 (11.8)	
Alemtuzumab	1 (3)^b^	1 (5.3)	0 (0)	
MOG IgG1 result prior to discontinuation, *n* (%)^c^				
Positive/low positive	151 (77.0)	98 (71.5)	53 (89.8)	0.005
Negative	45 (23.0)	39 (28.5)	6 (10.2)	
Reason for discontinuation, *n* (%)				
Planned discontinuation	210 (89.0)	136 (94.4)	74 (80.4)	0.008
Patient choice/non-compliance	18 (7.6)	5 (3.5)	13 (14.1)	
Adverse event	7 (3.0)	3 (2.1)	4 (4.3)	
Pregnancy planning	1 (0.4)	0 (0)	1 (1.1)	
Follow-up after discontinuation, years, median (IQR; range)	3.1 (1.3–6.3; 0.01–50.8)	2.1 (1.0–4.5; 0.01–11.6)	5.9 (2.6–8.7; 0.3–50.8)	<0.001
Time-to-post discontinuation relapse, months, median (IQR; range)	–	–	5.4 (1.4–20.1; 0.07–169.6)	–

IQR = interquartile range; IT = immunomodulatory treatment; IVIG = intravenous immunoglobulin.

^a^Of 36 IT intervals during which non-steroid IT was used, 4/36 intervals included exposure to two non-steroid IT (combinations of azathioprine and mycophenolate in two, azathioprine and IVIG in one, and mycophenolate and IVIG in one).

^b^One female patient with onset attack of brainstem and myelitis mixed phenotype followed by several myelitis relapses was initially diagnosed as multiple sclerosis and treated with alemtuzumab. Subsequently, her case was re-evaluated, and serum MOG IgG1 by live cell-based assay returned a clear positive. She fulfilled MOG antibody-associated disease (MOGAD) diagnostic criteria, and the final impression was that her presentation was most consistent with MOGAD. She has remained relapse-free over a 7-year period of follow-up after alemtuzumab.

^c^Available for 196/236 (83.1%) discontinuations.

### Predictors of relapse following treatment discontinuation

Relapse occurred following 92 (39.0%) discontinuations at a median time of 5.4 months (IQR 1.4–20.1) post-discontinuation. Those who relapsed were more likely to have a relapsing course at the time of discontinuation (50% versus 27.8%, *P* = 0.001) and a positive/low positive pre-discontinuation MOG IgG1 result (89.8% versus 71.5%, *P* = 0.005; Table 2). Median time from discontinuation to the last MOG IgG1 test was 4.4 months (IQR 1.7–8.3, range 0–90.7). Both the duration of IT and the duration between the last attack and discontinuation were shorter in those who relapsed (Table 2). These durations were highly correlated (Pearson correlation coefficient = 0.87), and IT duration was shorter (median difference of 0.6 months, IQR 0.3–1.2) because IT was started following the last attack in most cases.

In univariable analyses, longer IT durations were associated with reduced relapse hazard (Table 3 and Supplementary Fig. 2). A negative MOG IgG1 result was associated with lower hazard for post-discontinuation relapse [HR 0.43; 95% confidence interval (CI) 0.19–0.98; *P* = 0.045]. A relapsing course at the time of IT discontinuation doubled the relapse hazard compared to a monophasic course at the time of discontinuation (HR 2.02; 95% CI 1.35–3.01; *P* < 0.001). Early acute treatment of the onset attack had no effect on subsequent relapse risk.

**Table 3:** Predictors for post-immunomodulatory treatment discontinuation relapse by univariable Cox regression

Variable	HR (95% CI)	*P-*value
Age at onset, years	1.00 (0.99–1.01)	0.56
Sex		
Female	1 (Reference)	–
Male	1.02 (0.65–1.62)	0.92
Onset attack phenotype		
Optic neuritis	1 (Reference)	–
Myelitis	0.92 (0.47–1.83)	0.82
ADEM or brain	1.07 (0.46–2.46)	0.88
Mixed	1.04 (0.59–1.85)	0.89
Acute management at onset attack		
Early (<5 days)	1 (Reference)	–
Intermediate (5–14 days)	1.60 (0.72–3.54)	0.20
Late (>14 days) or no treatment	1.48 (0.69–3.19)	0.30
Duration of maintenance IT interval before discontinuation^a^		
1 month	1.31 (1.18–1.45)	<0.001
3 months	1 (Reference)	–
6 months	0.69 (0.60–0.80)	<0.001
12 months	0.42 (0.30–0.59)	<0.001
18 months	0.38 (0.26–0.55)	<0.001
Maintenance IT used		
Corticosteroids only	1 (Reference)	–
Non-steroid IT only	1.25 (0.54–2.92)	0.60
Combination of corticosteroids and non-steroid IT	1.29 (0.76–2.18)	0.34
Disease course at discontinuation		
Monophasic	1 (Reference)	–
Relapsing	2.02 (1.35–3.01)	<0.001
MOG IgG_1_ result prior to discontinuation		
Positive/low positive	1 (Reference)	–
Negative	0.43 (0.19–0.98)	0.045

ADEM = acute disseminated encephalomyelitis; CI = confidence interval; HR = hazard ratio; IT = immunomodulatory treatment; MOGAD = MOG antibody-associated disease.

^a^IT duration modelled as a restricted cubic spline. HR (95% CI) calculated for several discrete treatment durations with reference to 3 months.

**Figure 1:**
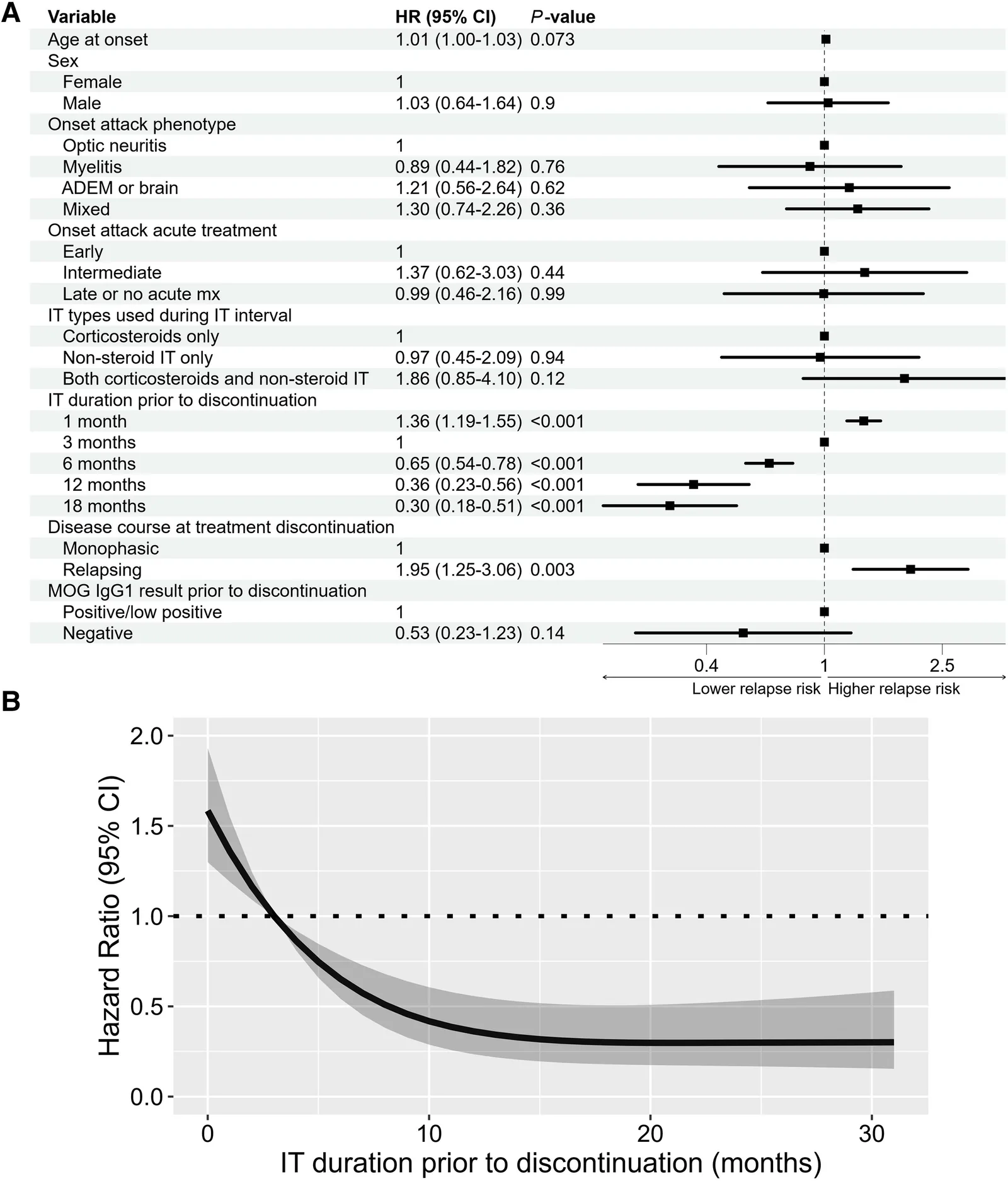
**Multivariable Cox regression for predictors of time-to-relapse after immunomodulatory treatment discontinuation for the overall cohort**. (**A**) Multivariable Cox regression results for all maintenance IT discontinuations. IT duration prior to discontinuation was modelled by a restricted cubic spline. Hazard ratios were calculated for several IT durations with reference to 3 months duration and reported in the forest plot. (**B**) Hazard ratio (with 95% confidence interval represented by the grey shaded band) was also plotted continuously as a function of IT duration. ADEM = acute disseminated encephalomyelitis; CI = confidence interval; HR = hazard ratio; IT = immunomodulatory treatment.

Multivariable analysis revealed overall consistent results (Fig. 1). Relapse hazard reduced with increasing maintenance IT duration (Fig. 1B). A relapsing course at the time of discontinuation doubled the relapse risk (HR 1.95; 95% CI 1.25–3.06; *P* = 0.003). A negative MOG IgG1 result was not significantly associated with relapse risk after covariate adjustment (HR 0.53; 95% CI 0.23–1.23; *P* = 0.14). The timing of acute treatment for the onset attack did not affect relapse risk.

In a sensitivity analysis which included 171 IT intervals with available MOG IgG1 status within 12 months prior to discontinuation, a negative MOG IgG1 result was significantly associated with reduced hazard for post-discontinuation relapse in both univariable (HR 0.34; 95% CI 0.14–0.87; *P* = 0.024) and multivariable (HR 0.38; 95% CI 0.15–0.99; *P* = 0.49) analyses. The associations of other variables remained similar to those in our main model.

### Discontinuations after a single onset attack

**Figure 2:**
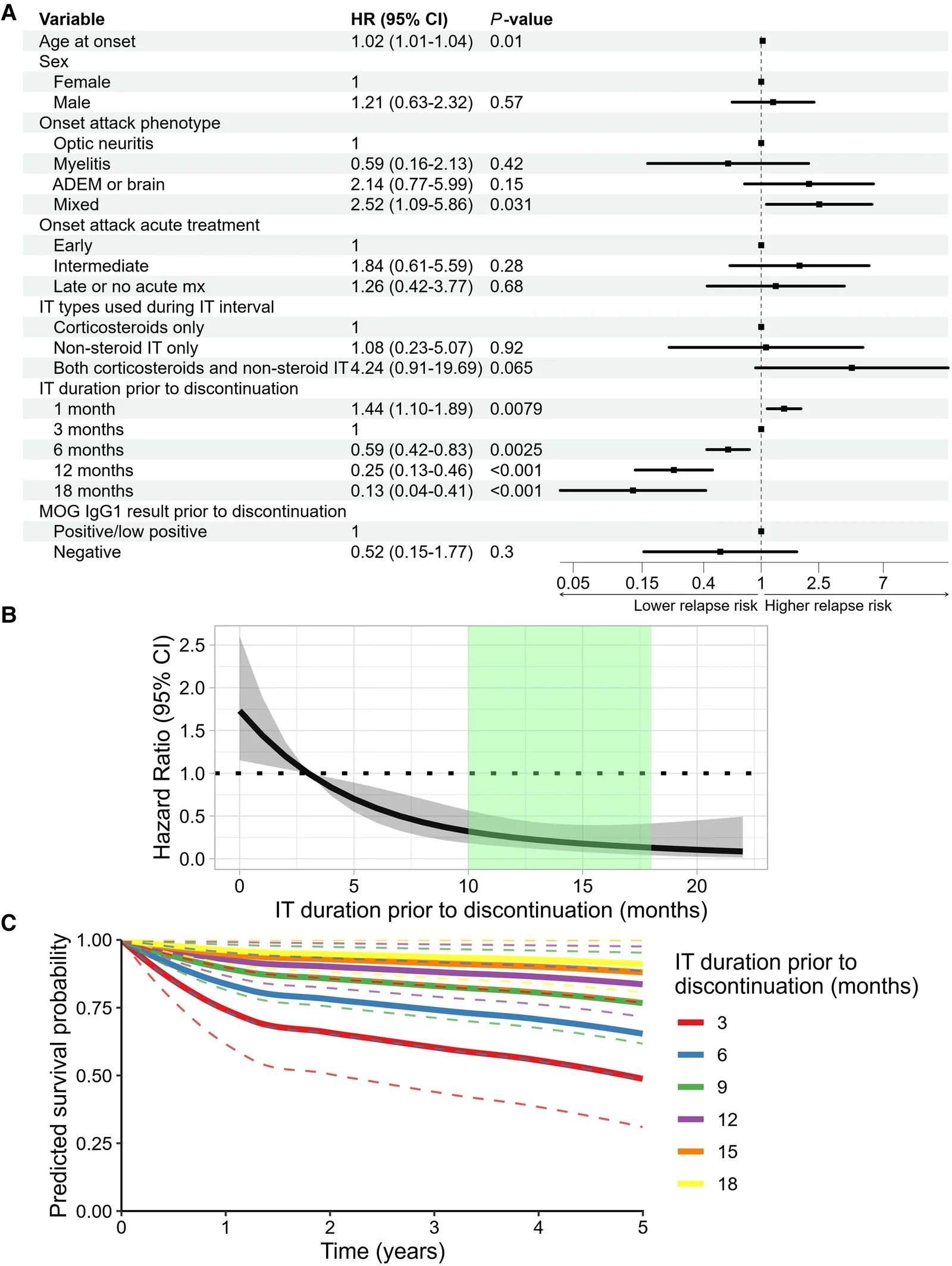
**Post-discontinuation relapse risk in patients with a monophasic course at time of discontinuation**. (**A**) Multivariable Cox regression was performed for predictors of time-to-relapse following IT discontinuation in patients who were treated after the onset attack with a monophasic course at time of discontinuation, and HR calculated for several IT durations (reference: 3 months duration). (**B**) Adjusted HR (95% CI represented by the grey shaded band) is plotted against IT duration (reference: 3 months), and an estimated optimal treatment duration of 10–18 months is identified (green band). (**C**) Relapse-free survival (95% CI represented by dashed lines) was calculated for specific IT durations; variables used for estimation were set at their medians as follows: sex, female; age at onset, 31.5 years; onset attack, optic neuritis; onset attack acute management, late or no acute management; IT used, corticosteroids only; and MOG IgG1 result, positive/low-positive. ADEM = acute disseminated encephalomyelitis; CI = confidence interval; HR = hazard ratio; IT = immunomodulatory treatment.

We analysed discontinuations in 150 patients with monophasic disease (i.e. a single attack and no relapse) at the time of IT discontinuation. In a multivariable analysis, treatment durations >3 months were associated with greater reductions in relapse risk (Fig. 2A). Visualizing the plot of adjusted HR by treatment duration shows a flattening gradient from 10–15 months onwards, with decreasing precision of estimates thereafter (Fig. 2B). Estimated survival curves show smaller differences in relapse-free survival between the curves for durations of 12–18 months compared with curves for durations under 12 months (Fig. 2C). Relapse-free survival at 2- and 5-years post-discontinuation was estimated at 92.8% (95% CI 86.0%–100%) and 87.7% (95% CI 77.0%–100%) for 15-month treatments and 65.5% (95% CI 50.1%–85.6%) and 47.9% (95% CI 29.9%–76.5%) for 3-month treatments prior to discontinuation. Based on these results, we estimated an optimal treatment duration of 10–18 months (Fig. 2B), which balances relapse hazard reduction and potential risk of chronic side effects from IT.

### Discontinuations in patients with a relapsing disease course

**Figure 3:**
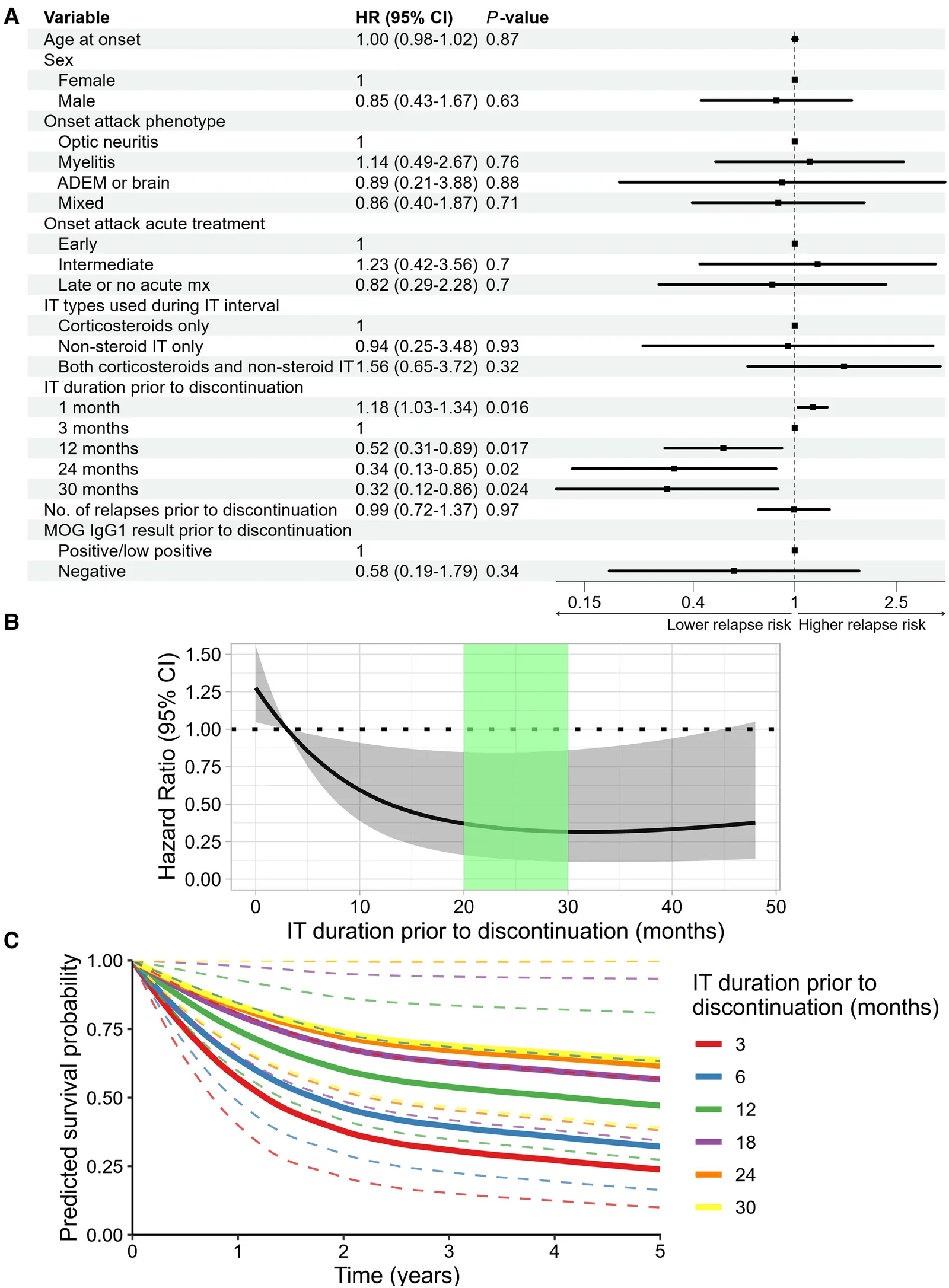
**Post-discontinuation relapse risk in patients with a relapsing course at time of discontinuation**. (**A**) Multivariable Cox regression was performed for predictors of time-to-relapse following IT discontinuation in patients with a relapsing course at time of discontinuation, and HR calculated for several IT durations (reference: 3 months duration). (**B**) Adjusted HR (95% CI represented by the grey shaded band) is plotted against IT duration (reference: 3 months) and an estimated optimal treatment duration of 20–30 months is identified (green band). (**C**) Relapse-free survival (95% CI represented by dashed lines) was calculated for specific IT durations; variables used for estimation were set at their medians as follows: sex, female; age at onset, 31.5 years; onset attack, optic neuritis; onset attack acute management, late or no acute management; IT used, corticosteroids only; MOG IgG1 result, positive/low positive; and number of relapses before time of discontinuation, 1. ADEM = acute disseminated encephalomyelitis; CI = confidence interval; HR = hazard ratio; IT = immunomodulatory treatment.

There were 86 IT discontinuations in 65 relapsing patients. The number of relapses before discontinuation was not associated with post-discontinuation relapse risk (Fig. 3A). Prolonged treatment durations were significantly protective against relapse relative to a 3-month duration. The effect of IT duration on relapse risk reduction plateaued beyond approximately 20 months (Fig. 3B). Survival curves demonstrated similar survival probabilities for treatment durations at and above 24 months (Fig. 3C). Two- and five-year relapse-free survival probabilities were estimated as 70.8% (95% CI 50.3%–99.6%) and 62.1% (95% CI 38.6%–99.9%) for 24 months of treatment, whereas these were 35.9% (95% CI 19.3%–66.5%) and 24.3% (95% CI 10.3%–57.1%) for 3 months of treatment. Considering these results together, we estimated an optimal treatment duration of 20–30 months (Fig. 3B), following which treatment discontinuation could be considered in patients with a relapsing disease course who had quiescent disease on treatment.

### Incident cohort analysis

We analysed our incident cohort to mitigate bias introduced by the enrichment of patients with a relapsing course, which may particularly affect patients who discontinued IT when their disease course was monophasic. There were 148 patients with 168 treatment discontinuations. At last follow-up, 44/148 (29.7%) patients had a relapsing course. For 133 discontinuations in patients in the incident cohort when their disease course was monophasic, the results of the multivariable analysis were similar. Again, a minimum treatment duration of approximately 10 months was significantly protective against relapse compared with 3 months (Supplementary Fig. 3).

## Discussion

In our observational study of patients with MOGAD who discontinued IT, we found that a prolonged IT course was effective at preventing relapse after discontinuation. Patients who had a relapsing course at the time they discontinued IT had a higher propensity for subsequent relapse than those who discontinued IT after their onset attack. Early acute treatment for the onset attack did not affect relapse risk. We estimated an optimal treatment duration in patients after a single attack of 10–18 months, which reduced post-discontinuation relapse risk by 67.9%–87.0% (relative to 3 months of treatment), with a predicted 5-year relapse-free survival of 87.7% for a 15-month course of IT compared to 47.9% for a 3-month course. For patients with a relapsing course who discontinued IT, we estimated an optimal duration of 20–30 months, which reduced post-discontinuation relapse risk by 63%–68.4%, with a predicted 5-year relapse-free survival rate of 62.1% for a 24-month course of IT.

The current treatment paradigm distinguishes between those who present with their first attack and those who develop relapsing disease. After the onset attack, patients have a 30%–50% probability of a relapsing course within 4–5 years, and so the benefit of long-term immunosuppression is not considered to outweigh the risks in most patients.^2,4,5,21^ Patients with proven relapsing propensity are, however, treated with long-term IT. Relapse rates are highest early after onset and decline over time.^2,4,9^ As such, IT discontinuation in stable patients has recently been proposed by several MOGAD experts to balance the risks of persistent immunosuppression and post-discontinuation relapse, respectively. However, the optimal treatment duration before discontinuation is not known, with a survey of experts revealing variable treatment practices, including the duration of steroid treatment after the first attack.^22^

Studies have sought to examine treatment duration after the onset attack and the effect on relapse risk. Several have investigated relapse from onset and dichotomized corticosteroid duration at a cut-off point of 5 weeks,^23^ 3 months^2,10^ or up to 6 months.^2,10^ A study with 109 patients investigated corticosteroid treatment duration and assessed multiple cut-off points.^3^ The authors showed that a course of at least 3 months (at ≥12.5 mg daily of prednisolone) was superior to <3 months for delaying time-to-relapse from onset, although the separation of survival curves appeared greater for longer periods. Only three studies have described relapse outcomes after discontinuation. In an earlier study from our group, which included up to 45 patients treated with IT after onset, relapse-free survival following discontinuation was higher in those treated for at least 3 months than for <3 months.^2^ A second study reported 21 patients who commenced IT after the first attack, with none relapsing after discontinuation.^24^ In a third study, 20 patients with a single attack discontinued IT after a median 15.4 months and remained relapse-free.^25^ Our study’s results are consistent in showing that a treatment period longer than 3 months is superior to 3 months in preventing relapse. Additionally, we showed this benefit on post-discontinuation relapse risk without including on-treatment time, as relapse suppression is expected while on IT. We estimate an optimal duration of 10–18 months, which is superior to 3 months in relapse prevention, based on our analyses and predictions, and we propose this as a reasonable balance between probabilities for remission induction and treatment side effects; however, this should be balanced according to the individual patient experience and views, as well as their comorbidities. As early relapses (within the first year) predict longer-term relapse, effective prevention of these may be important in preventing a chronic relapsing course.^26^

Data that guide discontinuation in relapsing patients are limited. One study described no relapses after discontinuation in seven relapsing patients.^24^ In another study, 10/21 (47.6%) relapsing patients experienced a relapse after discontinuation.^25^ Those who relapsed were treated for a shorter duration, consistent with our results. We also found that patients with a relapsing course when they discontinued IT had an elevated relapse risk compared to those who discontinued after a single onset attack. In these patients, our data and modelling suggest a minimum treatment duration of 20–30 months before considering discontinuation, although this decision should again be individualized based on the patient’s risk-benefit profiles, including consideration of their comorbidities, preferences and prior relapse characteristics such as severity. Because most patients were started on IT at the time of a relapse and only seven patients experienced an on-treatment relapse, IT treatment time was highly correlated with time stable on treatment and time since last relapse, with a median difference of <1 month.

Specific populations to consider when individualizing treatment decisions include the paediatric population and women of childbearing age. Although the relapse risk in young adults is greater than in children and older adults,^4,5^ women of childbearing age may prefer to take shorter treatment courses, especially because during pregnancy the relapse risk appears lower.^27^ Ideally, pregnancy should be planned and women with MOGAD counselled regarding relapse risks and treatment approaches, including IT compatibility with pregnancy and breastfeeding, during the peri-pregnancy period. In children, who have a lower relapse risk than young adults (but not older adults),^4,5^ some clinicians have a higher threshold before treating and may use shorter or no corticosteroids after an onset attack and/or because of concerns about corticosteroids affecting growth.^28^ In these instances, IVIG has been used instead of steroids. There is no consensus on whether to treat children and women of childbearing age less aggressively or similarly to other MOGAD patients, and these decisions should be individualized by considering risks and benefits of treatment approaches, as informed by our and other data.

Two studies have shown reduced relapse risk when acute treatment is initiated early after MOGAD onset, suggesting that very early treatment within days could ‘reset’ the immune system, whereas even a 5-day delay produces a less efficacious outcome.^12,23^ However, another study showed no difference in the effect on relapse risk of steroid treatment started <7 days from onset versus ≥7 days or no treatment.^3^ We did not identify early acute treatment initiation to modify post-IT discontinuation relapse risk. Differences between these studies may explain the divergent results, including different proportions of patients on background immunosuppression/corticosteroids, whether the analysis accounted for disease duration, and whether time on immunosuppression is included when baseline is set at onset rather than at time of IT discontinuation (as in our study). Nevertheless, early acute treatment remains important to optimize recovery from disability after an attack, as has been shown in optic neuritis related to MOGAD and NMOSD.^29-31^

Several studies have shown the prognostic value of MOG IgG status over time, with seroreversion to a ‘negative’ status associated with a reduced risk of relapse in univariable analyses.^13-15^ We were unable to confirm this in our multivariable analyses of relapse risk post-IT discontinuation, although univariable analysis showed seroreversion to be protective. One reason may be the variable timing of the last MOG IgG1 test relative to the time of IT discontinuation in our cohort, which reflects real-world practice and may have limited our ability to discern the predictive value of pre-discontinuation MOG antibody status. This is supported by our sensitivity analysis, which showed a significant protective effect with a negative MOG IgG1 result within 12 months before treatment discontinuation. However, in terms of practical utility, patients who serorevert are at very low risk, but this is not zero.^13,15^ Furthermore, the majority remain seropositive, despite many remaining relapse-free. The low risk of relapse, even in the MOG-IgG-positive patients, may have limited our ability to detect a significant predictive effect of MOG-IgG status in our total cohort.^15^ Overall, serial MOG-IgG status likely assists in decision-making regarding treatment discontinuation, though we would not use MOG-IgG status in isolation to decide whether to discontinue a relapsing MOGAD patient following a sustained period of disease control.

One patient in our cohort had completed treatment with alemtuzumab after several relapses and remained relapse-free across their post-treatment follow-up period of 7 years. It is worth noting that four reported cases of relapsing MOGAD treated with alemtuzumab showed failure to prevent additional relapse.^32,33^ Alemtuzumab leading to secondary antibody-associated autoimmunity is well-recognized and likely in part due to its immune reconstitution dynamics.^34-37^ Thus, alemtuzumab should not be used in treating MOGAD. Work to identify the most efficacious treatments for MOGAD, including whether treatments used in other autoimmune diseases may have a role in MOGAD management, remains important.

### Limitations and strengths

There are several limitations to this study. First, our observational study may have been affected by selection bias, although studies of this type are important for uncovering insights into rare diseases using real-world data. Additionally, observational studies allow multiple treatment periods that would not have been selected in a prospective study design. Using a single service cohort, in which patients are all seen by the same two expert NMOSD consultants, reduces between-centre and between-clinician heterogeneity and improves power to measure effects. We focused on patients with at least 1 year of follow-up, as we believed this was a clinically meaningful minimum period to capture relapse and treatment start/stop data. Selection bias may have been introduced if these patients’ follow-ups were associated with outcome, such as severe early fatal attacks. This was not the case in our cohort as (i) we observed 5/47 (10.6%) patients with <1 year follow-up experiencing relapse over a median 7.1 months; and (ii) the service setup is such that MOGAD patients, when reviewed through one of two highly specialized services, are not discharged and are followed up directly, remotely or via their neurologist for outcomes or through outreach clinics. Second, the variable timing of the pre-discontinuation MOG IgG1 test, which reflects real-world practice, may have led to underestimation of its predictive value. Third, most of our cohort had used oral steroids only, and we were not able to study specific outcomes after discontinuation of steroid-sparing agents as a sub-cohort or differences in outcomes of different ITs. The most common category of ‘steroids only’ as maintenance likely reflected the practice in the UK over the last decade or so, as well as patient choice to not start a steroid-sparing agent in some cases, although the reason for the choice of treatment was not specifically studied. Current practice is to commence a steroid-sparing agent in patients who are managed on oral steroids and are felt to benefit from a prolonged duration of immunomodulation to avoid long-term side effects of steroids. The relative efficacy of steroids and steroid-sparing agents is not known, with the choice of a steroid-sparing agent being empirical and informed by several observational studies. Fourth, our cohort was in the majority white, reflecting the population served, and we were unable to investigate race as a predictor of post-discontinuation relapse, given our sample size. Fifth, we did not have available MRI data on lesions or their evolution in this study.

## Conclusion

Our study demonstrates the benefit of a prolonged treatment course in reducing the subsequent risk of relapse in MOGAD patients who discontinue treatment. This applies to patients who have had a single attack and discontinue treatment when their disease course is monophasic, as well as patients who have a relapsing course and discontinue treatment, although the latter are at higher risk for post-discontinuation relapse. We propose that, in patients who intend to discontinue IT and have had a single attack, IT be continued for at least 10–18 months, with no on-treatment relapse before discontinuation, to optimize the probability of inducing disease remission. In relapsing patients, a more prolonged treatment course of at least 20–30 months, with no on-treatment relapse, should be attained before consideration of treatment cessation. These data help to inform decisions around IT discontinuation as a strategy that balances the risks of relapse and the complications of chronic immunomodulatory treatment.

## Supplementary Material

awag006_Supplementary_Data

## Data Availability

The anonymized grouped data that support the findings of this study are available from the corresponding author to qualified non-commercial investigators, on reasonable request and in accordance with consent and ethics requirements.
