# Supplementary material for: Optimal strategies for treatment discontinuation in MOG antibody-associated disease
Source: Brain. 2026 Feb 4;149(9):3242–53. doi: 10.1093/brain/awag006 (PMC13548856; doi:10.1093/brain/awag006)
Supplement: Supplementary file 1 [file awag006_supplementary_data.pdf]

Supplementary material

Yeh WZ, Francis A, Cooper S, et al. Optimal Strategies for Treatment Discontinuation in Myelin Oligodendrocyte Glycoprotein Antibody-Associated Disease

Supplementary Fig. 1: Flowchart of Participant Inclusion 2

Supplementary Fig. 2: Univariable Analysis of Time-To-Relapse by Maintenance Immunomodulatory Treatment Duration 3

Supplementary Fig. 3: Patients of the Incident Cohort With a Monophasic Course at Time of Discontinuation and Treatment Duration 4

Supplementary Fig. 1: Flowchart of Participant Inclusion

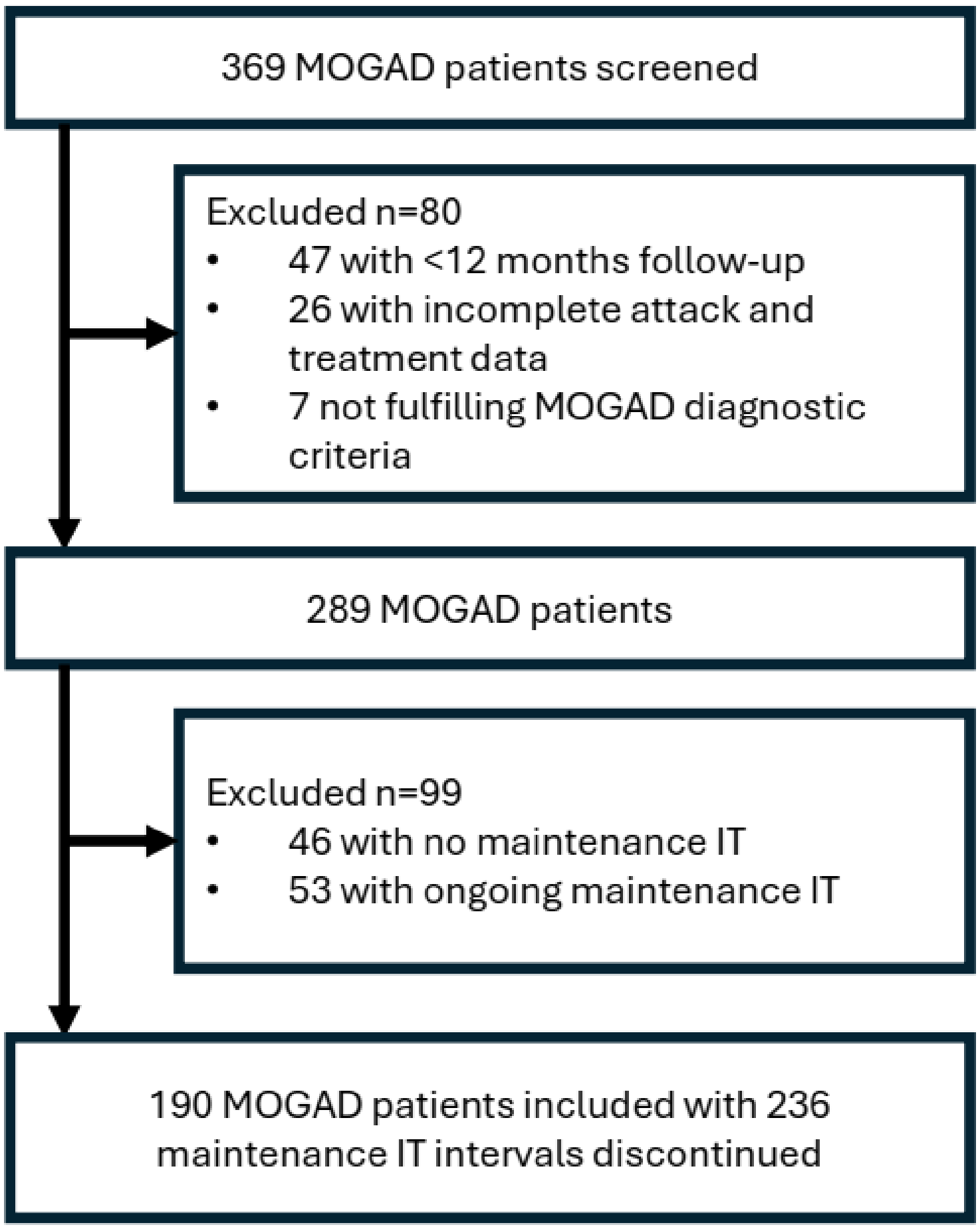

IT, immunomodulatory treatment; MOG, myelin oligodendrocyte glycoprotein; MOGAD, myelin oligodendrocyte glycoprotein antibody-associated disease

Supplementary Fig. 2: Univariable Analysis of Time-To-Relapse by Maintenance Immunomodulatory Treatment Duration

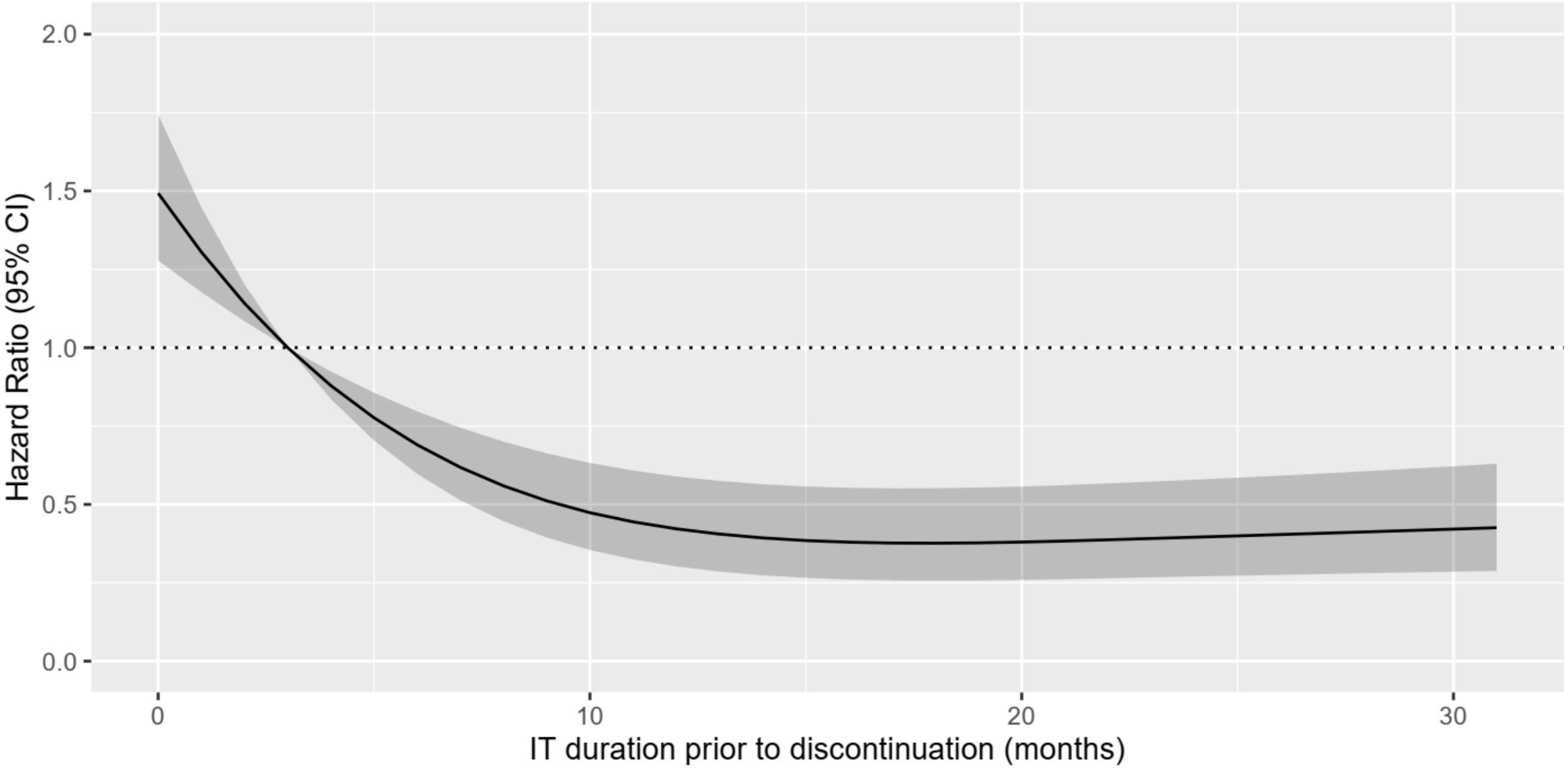

Hazard ratio for time-to-relapse after immunomodulatory treatment (IT) discontinuation plotted as function of duration of IT prior to discontinuation. IT, immunomodulatory treatment.

Supplementary Fig. 3: Patients of the Incident Cohort With a Monophasic Course at Time of Discontinuation and Treatment Duration

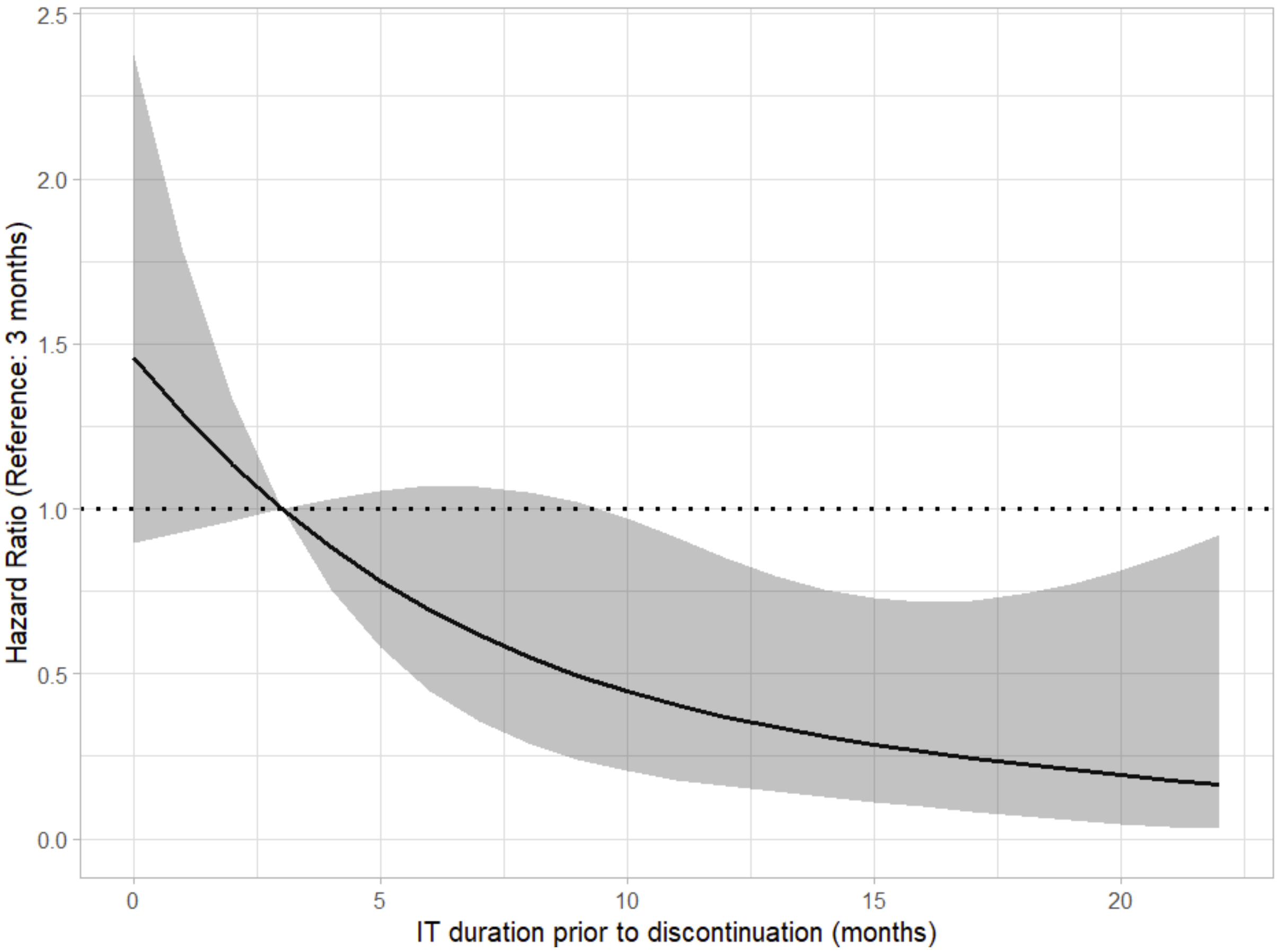

Multivariable Cox regression analysis was performed and adjusted HR (95% CI represented by grey band) plotted against IT duration. CI, confidence interval; HR, hazard ratio; IT, immunomodulatory treatment.
